# Supplementary material for: Modeling glioblastoma heterogeneity as a dynamic network of cell states
Source: Mol Syst Biol. 2021 Sep 16;17(9):e10105. doi: 10.15252/msb.202010105 (PMC8444284; doi:10.15252/msb.202010105)
Supplement: Supplementary file 5 — Source Data for Figure 3 [file MSB-17-e10105-s001.zip › Figure3A_sourcedata/GSEA_3065/nosigdb_state4.GseaPreranked.1622718430994/RICHARDS_ZHONG_NATURE_2018_NPCS_UPREG.html]

Details for gene set RICHARDS\_ZHONG\_NATURE\_2018\_NPCS\_UPREG[GSEA]

|  || Dataset | state4 |
| Phenotype | NoPhenotypeAvailable |
| Upregulated in class | na\_neg |
| GeneSet | RICHARDS\_ZHONG\_NATURE\_2018\_NPCS\_UPREG |
| Enrichment Score (ES) | -0.8591904 |
| Normalized Enrichment Score (NES) | -3.175274 |
| Nominal p-value | 0.0 |
| FDR q-value | 0.0 |
| FWER p-Value | 0.0 |
Table: GSEA Results Summary

  

Fig 1: Enrichment plot: RICHARDS\_ZHONG\_NATURE\_2018\_NPCS\_UPREG      
 Profile of the Running ES Score & Positions of GeneSet Members on the Rank Ordered List

  

| PROBE | GENE SYMBOL | GENE\_TITLE | RANK IN GENE LIST | RANK METRIC SCORE | RUNNING ES | CORE ENRICHMENT || 1 | SOX9 |  |  | 48 | 0.299 | -0.0005 | No |
| 2 | RPS27L |  |  | 149 | 0.223 | -0.0113 | No |
| 3 | HSPB1 |  |  | 180 | 0.211 | -0.0110 | No |
| 4 | TAGLN2 |  |  | 672 | 0.118 | -0.0880 | No |
| 5 | ZFHX4 |  |  | 744 | 0.109 | -0.0969 | No |
| 6 | VIM |  |  | 1350 | 0.069 | -0.1937 | No |
| 7 | HES1 |  |  | 1442 | 0.065 | -0.2069 | No |
| 8 | GLI3 |  |  | 1603 | 0.059 | -0.2315 | No |
| 9 | TGIF1 |  |  | 1699 | 0.055 | -0.2457 | No |
| 10 | GNG5 |  |  | 1709 | 0.055 | -0.2458 | No |
| 11 | UCP2 |  |  | 1737 | 0.054 | -0.2488 | No |
| 12 | TUBA1B |  |  | 3658 | 0.007 | -0.5612 | No |
| 13 | PLIN2 |  |  | 4233 | -0.024 | -0.6540 | No |
| 14 | NOTCH1 |  |  | 4321 | -0.031 | -0.6674 | No |
| 15 | ID4 |  |  | 4329 | -0.031 | -0.6678 | No |
| 16 | TMEM98 |  |  | 4455 | -0.041 | -0.6871 | No |
| 17 | MDK |  |  | 5110 | -0.093 | -0.7913 | No |
| 18 | RNASEH2A |  |  | 5241 | -0.105 | -0.8099 | No |
| 19 | H2AFV |  |  | 5381 | -0.117 | -0.8297 | No |
| 20 | C21orf58 |  |  | 5382 | -0.117 | -0.8268 | No |
| 21 | WDR34 |  |  | 5484 | -0.130 | -0.8401 | No |
| 22 | DEPDC1B |  |  | 5570 | -0.138 | -0.8505 | No |
| 23 | BUB3 |  |  | 5583 | -0.141 | -0.8490 | No |
| 24 | HMGB3 |  |  | 5584 | -0.142 | -0.8455 | No |
| 25 | CENPW |  |  | 5585 | -0.142 | -0.8421 | No |
| 26 | KNSTRN |  |  | 5610 | -0.146 | -0.8424 | No |
| 27 | CREB5 |  |  | 5611 | -0.146 | -0.8388 | No |
| 28 | MCM6 |  |  | 5644 | -0.151 | -0.8403 | No |
| 29 | CLIC1 |  |  | 5664 | -0.154 | -0.8397 | No |
| 30 | MCM2 |  |  | 5785 | -0.180 | -0.8548 | Yes |
| 31 | HAT1 |  |  | 5803 | -0.184 | -0.8530 | Yes |
| 32 | PRC1 |  |  | 5835 | -0.192 | -0.8534 | Yes |
| 33 | PAICS |  |  | 5858 | -0.196 | -0.8522 | Yes |
| 34 | CDCA4 |  |  | 5861 | -0.197 | -0.8477 | Yes |
| 35 | CDCA7 |  |  | 5863 | -0.198 | -0.8430 | Yes |
| 36 | CKAP2 |  |  | 5879 | -0.201 | -0.8405 | Yes |
| 37 | C19orf48 |  |  | 5894 | -0.205 | -0.8378 | Yes |
| 38 | BTG3 |  |  | 5895 | -0.206 | -0.8327 | Yes |
| 39 | SPAG5 |  |  | 5899 | -0.207 | -0.8282 | Yes |
| 40 | SNRPB |  |  | 5908 | -0.209 | -0.8243 | Yes |
| 41 | PRR11 |  |  | 5926 | -0.214 | -0.8218 | Yes |
| 42 | RCN1 |  |  | 5945 | -0.219 | -0.8194 | Yes |
| 43 | KIF2C |  |  | 5996 | -0.237 | -0.8218 | Yes |
| 44 | ITGB3BP |  |  | 6005 | -0.240 | -0.8172 | Yes |
| 45 | RPA3 |  |  | 6014 | -0.244 | -0.8125 | Yes |
| 46 | FEN1 |  |  | 6035 | -0.256 | -0.8095 | Yes |
| 47 | BUB1 |  |  | 6045 | -0.266 | -0.8044 | Yes |
| 48 | HJURP |  |  | 6055 | -0.271 | -0.7992 | Yes |
| 49 | LDHA |  |  | 6057 | -0.272 | -0.7927 | Yes |
| 50 | MCM5 |  |  | 6069 | -0.278 | -0.7877 | Yes |
| 51 | SPC25 |  |  | 6072 | -0.279 | -0.7812 | Yes |
| 52 | GMNN |  |  | 6080 | -0.285 | -0.7754 | Yes |
| 53 | FBXO5 |  |  | 6084 | -0.287 | -0.7688 | Yes |
| 54 | KIF22 |  |  | 6089 | -0.290 | -0.7624 | Yes |
| 55 | CENPN |  |  | 6090 | -0.291 | -0.7553 | Yes |
| 56 | NUF2 |  |  | 6093 | -0.293 | -0.7484 | Yes |
| 57 | CDCA5 |  |  | 6099 | -0.296 | -0.7420 | Yes |
| 58 | CCNA2 |  |  | 6100 | -0.297 | -0.7347 | Yes |
| 59 | DUT |  |  | 6107 | -0.303 | -0.7283 | Yes |
| 60 | CKAP2L |  |  | 6108 | -0.303 | -0.7208 | Yes |
| 61 | KIF11 |  |  | 6109 | -0.304 | -0.7134 | Yes |
| 62 | MCM3 |  |  | 6113 | -0.307 | -0.7063 | Yes |
| 63 | MELK |  |  | 6117 | -0.312 | -0.6992 | Yes |
| 64 | MIS18BP1 |  |  | 6122 | -0.316 | -0.6921 | Yes |
| 65 | PHGDH |  |  | 6126 | -0.318 | -0.6848 | Yes |
| 66 | TACC3 |  |  | 6138 | -0.328 | -0.6785 | Yes |
| 67 | CCNB2 |  |  | 6140 | -0.329 | -0.6706 | Yes |
| 68 | NDC80 |  |  | 6142 | -0.330 | -0.6627 | Yes |
| 69 | AURKB |  |  | 6143 | -0.332 | -0.6546 | Yes |
| 70 | RRM1 |  |  | 6147 | -0.338 | -0.6468 | Yes |
| 71 | CDC20 |  |  | 6154 | -0.344 | -0.6393 | Yes |
| 72 | DLGAP5 |  |  | 6156 | -0.345 | -0.6310 | Yes |
| 73 | KIF15 |  |  | 6158 | -0.346 | -0.6227 | Yes |
| 74 | CDKN3 |  |  | 6160 | -0.349 | -0.6143 | Yes |
| 75 | KIF23 |  |  | 6162 | -0.351 | -0.6059 | Yes |
| 76 | HMGN2 |  |  | 6163 | -0.351 | -0.5973 | Yes |
| 77 | CDCA3 |  |  | 6165 | -0.355 | -0.5888 | Yes |
| 78 | ANP32E |  |  | 6168 | -0.357 | -0.5803 | Yes |
| 79 | CENPK |  |  | 6169 | -0.363 | -0.5714 | Yes |
| 80 | MCM4 |  |  | 6171 | -0.372 | -0.5625 | Yes |
| 81 | PCNA |  |  | 6173 | -0.374 | -0.5535 | Yes |
| 82 | DTYMK |  |  | 6177 | -0.381 | -0.5446 | Yes |
| 83 | CENPM |  |  | 6179 | -0.386 | -0.5354 | Yes |
| 84 | GINS2 |  |  | 6194 | -0.420 | -0.5274 | Yes |
| 85 | ZWINT |  |  | 6195 | -0.425 | -0.5170 | Yes |
| 86 | TUBB4B |  |  | 6196 | -0.431 | -0.5064 | Yes |
| 87 | UHRF1 |  |  | 6198 | -0.436 | -0.4959 | Yes |
| 88 | RRM2 |  |  | 6199 | -0.438 | -0.4851 | Yes |
| 89 | DHFR |  |  | 6200 | -0.441 | -0.4743 | Yes |
| 90 | PBK |  |  | 6207 | -0.465 | -0.4639 | Yes |
| 91 | BIRC5 |  |  | 6214 | -0.484 | -0.4531 | Yes |
| 92 | GTSE1 |  |  | 6217 | -0.490 | -0.4414 | Yes |
| 93 | CLSPN |  |  | 6218 | -0.490 | -0.4294 | Yes |
| 94 | SMC4 |  |  | 6219 | -0.490 | -0.4174 | Yes |
| 95 | HELLS |  |  | 6221 | -0.501 | -0.4053 | Yes |
| 96 | NUSAP1 |  |  | 6223 | -0.507 | -0.3930 | Yes |
| 97 | SMC2 |  |  | 6229 | -0.518 | -0.3811 | Yes |
| 98 | TMPO |  |  | 6233 | -0.522 | -0.3688 | Yes |
| 99 | CKS1B |  |  | 6234 | -0.523 | -0.3560 | Yes |
| 100 | KPNA2 |  |  | 6236 | -0.543 | -0.3429 | Yes |
| 101 | H2AFX |  |  | 6237 | -0.576 | -0.3287 | Yes |
| 102 | ORC6 |  |  | 6238 | -0.585 | -0.3144 | Yes |
| 103 | CKS2 |  |  | 6239 | -0.585 | -0.3001 | Yes |
| 104 | UBE2T |  |  | 6240 | -0.593 | -0.2855 | Yes |
| 105 | CCNB1 |  |  | 6242 | -0.606 | -0.2709 | Yes |
| 106 | ATAD2 |  |  | 6243 | -0.607 | -0.2560 | Yes |
| 107 | PTTG1 |  |  | 6245 | -0.622 | -0.2409 | Yes |
| 108 | CDK1 |  |  | 6246 | -0.627 | -0.2256 | Yes |
| 109 | MAD2L1 |  |  | 6247 | -0.628 | -0.2102 | Yes |
| 110 | CENPU |  |  | 6248 | -0.633 | -0.1947 | Yes |
| 111 | H2AFZ |  |  | 6250 | -0.651 | -0.1789 | Yes |
| 112 | MCM7 |  |  | 6252 | -0.665 | -0.1628 | Yes |
| 113 | TPX2 |  |  | 6253 | -0.667 | -0.1464 | Yes |
| 114 | ASPM |  |  | 6254 | -0.680 | -0.1298 | Yes |
| 115 | UBE2C |  |  | 6255 | -0.750 | -0.1114 | Yes |
| 116 | HMGB2 |  |  | 6258 | -0.766 | -0.0930 | Yes |
| 117 | TOP2A |  |  | 6260 | -0.814 | -0.0732 | Yes |
| 118 | MKI67 |  |  | 6261 | -0.882 | -0.0516 | Yes |
| 119 | HIST1H4C |  |  | 6262 | -1.038 | -0.0262 | Yes |
| 120 | CENPF |  |  | 6263 | -1.068 | -0.0000 | Yes |
Table: GSEA details [plain text format]

  

Fig 2: RICHARDS\_ZHONG\_NATURE\_2018\_NPCS\_UPREG: Random ES distribution      
 Gene set null distribution of ES for **RICHARDS\_ZHONG\_NATURE\_2018\_NPCS\_UPREG**
